# Are Women Worse Than Men?

**Published:** 1895-06-15

**Authors:** 


### Are Women Worse Than Men?

The critics of Dr. Lombroso's book on the female offender has generally assumed that he is given to wholesale condemnation of womankind. The sensitive indignation that has been shown on the subject is a little unworthy of the claims to sweet reasonableness put forth by the women of our day, and is more than a little unjust. In the first place, Dr. Lombroso was speaking of criminal women, not of criminal men, whom he had already dealt with in "*L'Uomo Delinquente*"; and, in the second, he distinctly says that crime is rarer among women than among men. Moreover, his measurements show, as one of the steady characteristics of the criminal woman, a masculine strength of jaw and various features which the opinion of generations has defined as male. Indeed, he regards the instinct of degenerate woman as leading to prostitution rather than to crime, although in the criminal woman the first is usually the concomitant of the second. Very rarely do we find a woman who has led an honourable and moral life in the prisoner's dock, and when we do it is usually through some act which, though on the face of it criminal, springs as much from a disordered reason as from moral weakness. To this class belong those not infrequent cases where a mother kills her children. Occasionally a murder of this kind is due to clear insanity; but more frequently it springs from anxiety so acute that it verges on unreason. The woman is poor, and does not know how to get food for her children; or she is ill, and fears that if she dies they will be harshly treated, so it seems to her a justifiable thing to remove them once for all from the troubles of life. Such crimes are due to lack of courage or to weakness of intellect rather than to a true criminal instinct. Hysterical such women usually are, and the disease may lead them to suicide as easily as to murder, but they show none of the malice and cruelty, though they possess some of the recklessness, of the genuine criminal.

The born criminal, male or female, requires few incitements to crime. They are as susceptible to vanity and greed as a child, as instinctively given to rob and kill as any beast of prey. But as the fox must secure his prey in a different way from the lion, the woman, being weak, must gain her ends in a different way from the man who is strong. Not that acts of violence are wanting in the record of female crime, where the woman has had the strength to strike and slay; but generally her sins have been accomplished by means of subtlety, and a malicious ingenuity, which we cannot but regard with greater horror than we feel for the crimes of impulse, due to hot blood, and sometimes to intoxication. There is in the crimes of women a cruelty and deliberation that surpasses the bounds of men's capacity. Brinvilliers visited the hospitals, and under the semblance of charity, giving dainties to the poor and suffering, tested on those unhappy creatures, towards whom she felt not the slightest personal ill-will, the poisons she meant to use on those she hated, or whose death could benefit her. Some murderesses, unable through weakness to strike the blow themselves, hire a lover or bribe an assassin to commit the crime, and in such cases plan the circumstances of it with diabolical forethought.

Yet we find this very forethought entangled with folly in a way which indicates that the true impulse to crime is akin to madness. One poisoner brought suspicion on herself by saying, when first the drugs began to take effect on her victim, "He will die; he cannot recover," although as yet no serious symptoms had shown themselves, and even predicting the symptoms that would ensue. Madame Lafarge, having administered poison to her husband, went about pretending a superstitious fear of receiving a black-bordered letter, and began to ask how long it was customary for widows to wear mourning. Moreover, women who have the true criminal instinct rob, lie, and murder when, by the exercise of a little tact and patience, they might obtain their desires without crime. In *Blackwood's Magazine* there was told, a year or two ago, a story of a girl who repeatedly set fire to houses in her neighbourhood in order that the attention of the village might be distracted while she went to meet her lover. Whether the tale was founded on fact or not, it shows well the unreasonableness, the supreme egotism, of the born criminal. One woman, as chronicled by Lombroso, wishing to marry a man whom her parents disapproved of, murdered them, although in a few months she would have attained her majority, and been able to dispense with their consent. The readiness with which a woman with the criminal instinct will kill a husband or lover of whom she is tired is one of the characteristics which remove her farthest from the level of ordinary humanity. Another is the frequent absence of maternal instinct. Where she does not love the father she hates and maltreats the child, while the instinct of the average woman is to love the child the more and cling to it as a compensation when disappointed in her husband.

Yet there are instances in which a distorted maternal affection leads to crime. Under this head we may reckon such cases of child murder as we have already alluded to. But there is a far more common form of infanticide condoned in many civilised countries by public opinion. Very many women who would cherish a child who has once lain in their arms, have no hesitation in taking the life of the unborn. This springs usually, as we have said, from a distorted form of the maternal instinct. Where the mother feels that there are already enough mouths to fill, she shrinks from adding to the number. Above all is this the case when she herself is wholly, or in part, the bread-winner. In that case the time which must be devoted to the care of another child seems to be a robbery of those already existing. The crime is an almost inevitable result of a complicated social system, and is one of the chief condemnations to be pronounced against women entering public life, either industrial or social. For the *femme sole* there is no work unfitted for her for which she herself is fit, but the married woman has chosen wifehood and motherhood as her profession, and should undertake no work which cannot be made subsidiary to home duties. It is the neglect of this natural, and therefore inflexible, law which leads to the creation of such criminals as we speak of. But when one comes to the question of preventing such sins one trespasses on that great question of the marriage relation itself, concerning which so much has been said and written of late, that one is glad to fall back on the more probably wholesome effects of a little silence.

